# PSYCHOMETRIC PROPERTIES OF THE DANISH VERSION OF THE RESILIENCE SCALE FOR ADULTS IN INDIVIDUALS WITH ACQUIRED BRAIN OR SPINAL CORD INJURY, AND THEIR FAMILY MEMBERS

**DOI:** 10.2340/jrm.v57.44078

**Published:** 2025-10-29

**Authors:** Karoline Yde ANDERSEN, Anne NORUP, Mia Moth WOLFFBRANDT, Fin BIERING-SØRENSEN, Juan Carlos ARANGO-LASPRILLA, Pernille Langer SOENDERGAARD, Frederik Have DORNONVILLE DE LA COUR

**Affiliations:** 1 Neurorehabilitation Research and Knowledge Centre, Copenhagen University Hospital – Rigshospitalet, Glostrup; 2 Department of Neuroscience, University of Copenhagen, Copenhagen; 3 Department of Clinical Medicine, University of Copenhagen, Copenhagen; 4 Department of Brain and Spinal Cord Injuries, Rigshospitalet, Glostrup, Denmark; 5 Department of Cell Biology and Histology, University of the Basque Country UPV/EHU, Leioa; 6 IKERBASQUE, Basque Foundation for Science, Bilbao, Spain; 7 Neurorehabilitation-CPH, City of Copenhagen, Hellerup, Denmark; 8 The Elsass Foundation, Charlottenlund, Denmark

**Keywords:** caregivers, psychological tests, resilience, psychological, stroke, validation study

## Abstract

**Objective:**

To investigate validity and reliability of the Danish version of the Resilience Scale for Adults among individuals with acquired brain or spinal cord injury, and their family members.

**Design:**

Cross-sectional study.

**Subjects/Patients:**

Adults with acquired brain or spinal cord injury, and their family members.

**Methods:**

Unidimensionality, floor/ceiling effects, and internal consistency of the 6 subscales were analysed using confirmatory factor analysis. A series of models were estimated to investigate structural validity, and construct validity was analysed using correlations.

**Results:**

*Family cohesion*, *Planned future*, and *Perception of self* showed good reliability (ω = 0.79–0.83) and good model fit (Comparative fit index: 0.986–1.000). *Social resources* and *Social competence* demonstrated adequate reliability (ω = 0.81 and 0.75) and good fit, accounting for local dependency. *Structured style* had poor reliability (ω = 0.53) and model misfit. The Resilience Scale for Adults was best represented by a 6-factor correlated model, compared with a single first- or second-order factor, but all models showed inadequate fit. All scale scores correlated negatively with scores of anxiety and depression.

**Conclusion:**

All but 1 subscale demonstrated good psychometric properties. The Resilience Scale for Adults can be used to measure different aspects related to resilience for this mixed population.

## LAY ABSTRACT

Acquiring a brain or spinal cord injury can have considerable impact on the person with the injury and their family members. Resilience is defined as a person’s ability to adapt and recover after facing challenges, stress, or trauma. This study examined how well the Danish version of the Resilience Scale for Adults works for people with acquired brain injury, spinal cord injury, and their family members. The scale was tested for its reliability and accuracy in measuring resilience. The Resilience Scale for Adults showed good reliability overall, but one subscale, *Structured style*, did not perform well. People who scored higher on resilience reported fewer symptoms of anxiety and depression. The Danish version of the Resilience Scale for Adults is a reliable tool for understanding resilience in individuals affected by acquired brain injury or spinal cord injury and their family members, except for the subscale *Structured style*.

Acquired brain injury (ABI) and spinal cord injury (SCI) are severe neurological conditions that have a number of short- and long-term consequences not only for the person with the injury, but also for their family (1). Many studies have investigated the impact of the adverse consequences of an injury on the family (2–4), but only a few have examined mediating factors that buffer against experienced burden or distress (5). Adapting to new life circumstances and overcoming emotional and physical distress can depend on individual coping style, personality, and resilience (6–8). Resilience can be defined as the capacity to adapt positively to change, and it is a multidimensional concept extending beyond individual characteristics, abilities, and skills (8). It encompasses personal resources and support provided by family and social networks (9). High resilience has been associated with higher quality of life (10), higher self-efficacy (11), and fewer symptoms of depression and stress (12, 13) in individuals with ABI and SCI. Family members of individuals with ABI or SCI reporting high resilience show a positive correlation with emotional well-being and reduced caregiver burden (14, 15).

Studies examining the psychometric properties of resilience instruments are lacking for individuals with ABI and SCI. A methodological review evaluated the psychometric properties of 15 different resilience measures in non-patient populations and found that the Resilience Scale for Adults (RSA) was 1 of the 4 psychometrically stronger scales (16). The scale was developed by Friborg, Hjemdal, and colleagues and consisted originally of 45 items (9, 17). To improve reliability, the number of items was reduced to 33 across 6 subscales (9, 17–19). The scale has been validated in both a clinical and a non-clinical sample (9). Previous studies with samples of university students and individuals on long-term sick leave have shown good internal consistency with Cronbach’s alpha values ranging from 0.63–0.78 (20) and 0.50–0.86 (21), where the subscale *Structured style* showed lowest internal consistency. Construct validity has been tested, with negative correlations with measures of loneliness in university students (22) and anxiety and depression symptoms in individuals on long-term sick leave (21), and positive correlations with sense of coherence in university students (23). The scale has been translated into several languages and validated in cross-cultural studies (20, 23–25).

The psychometric properties of the Danish version of the RSA have only been examined in a sample of 575 university students (22). Therefore, the present study aimed to investigate the psychometric properties of the RSA in a combined sample of adults with ABI or SCI, and their family members. The purpose of this study was (*i*) to investigate the psychometric properties for the RSA subscales including unidimensionality, internal consistency, and floor and ceiling effect; (*ii*) to determine the factor structure for the total scale; and (*iii*) to evaluate convergent validity through correlations between subscales, and divergent validity through correlations with anxiety and depression symptoms.

## METHODS

This cross-sectional study is a secondary analysis of baseline data collected in a randomized controlled trial (RCT) of a family intervention in Denmark (26). Participants were enrolled from October 2018 to June 2021. The total sample consisted of 157 participants: individuals with ABI (*n* = 53) or SCI (*n* = 20), and their family members (*n* = 84). Individuals with ABI or SCI were recruited from 2 highly specialized neurorehabilitation departments in the eastern part of Denmark, between 6 months and 2 years after discharge (27). The population consisted of both traumatic and non-traumatic injuries. Each participant with an injury was accompanied by at least 1 family member (spouse, partner, adult children, parent, or siblings) and was enrolled if the following criteria were met: (*i*) ≥ 18 years of age, (*ii*) able to understand and speak Danish, and (*iii*) cognitively able to participate. Participants were excluded if they had previously been diagnosed with another neurological or psychiatric disorder or had experienced violence or substance abuse in their family. Details regarding inclusion and procedures related to the RCT are describ-ed elsewhere (26, 27). All participants completed the questionnaire independently. If needed, assistance was provided from a research nurse to ensure comprehension and completion of the questionnaire. Prior to analysis, 17 participants were excluded for not completing the RSA, resulting in a sample of 140 participants: 61 individuals with an injury (ABI or SCI) and 79 family members. Participant characteristics are described in Table I. Data from the RCT were collected at baseline before randomization.

**Table I:** Characteristics of participants

Characteristics	Total *n* = 140	Individuals with injury *n* = 61	Family members *n* = 79	*p*-value^e^
Sex, *n* (%)				
Female	69 (49%)	20 (33%)	49 (62%)	0.001
Male	71 (51%)	41 (67%)	30 (38%)	
Age				
Mean (SD)	51.2 (15.9)	50.7 (16.5)	51.6 (15.4)^a^	0.75
Range	18.2; 84.5	19.9; 84.5	18.2; 82.3	
Type of injury, *n* (%)				
TBI		20 (33%)		
Stroke		19 (31%)		
SCI		19 (31%)		
Other^b^		3 (5%)		
Time since injury^c^				
Mean (SD)		16.1 (8.76)		
Range		6;49		
Education, *n* (%)				
Low	44 (31%)	21 (34%)	23 (29%)	0.33
High^d^	91 (65%)	36 (59%)	55 (70%)	
Other	4 (3%)	3 (5%)	1 (1%)	
Missing	1 (1%)	1 (2%)	0	
Work status, *n* (%)				
Full-time occupation	71 (51%)	24 (39%)	47 (59%)	0.003
Student	10 (7%)	3 (5%)	7 (9%)	
Homemaker	3 (2%)	1 (2%)	2 (3%)	
Unemployed	5 (4%)	2 (3%)	3 (4%)	
Retired	29 (21%)	13 (21%)	16 (20%)	
Sick leave	19 (14%)	16 (26%)	3 (4%)	
Missing	3 (2%)	2 (3%)	1 (1%)	
Civil status, *n* (%)				
In a relationship	120 (86%)	49 (80%)	71 (90%)	0.20
Single	15 (11%)	8 (13%)	7 (9%)	
Other	3 (2%)	3 (5%)	0	
Missing	2 (1%)	1 (2%)	1 (1%)	
Living status, *n* (%)				
Living with partner or other	130 (93%)	54 (88%)	76 (96%)	0.17
Living alone	9 (6%)	6 (10%)	3 (4%)	
Missing	1 (1%)	1 (2%)	0	

a *n* = 76;

b tumour, heart attack, and haematological disease;

c reported in months;

d high level of education indicates a college or university degree;

e *t*-test was used for continuous variables, Fisher’s exact test was used for categorical variables.

In accordance with the Helsinki Declaration, all participants provided written informed consent. The study was reported using COSMIN Reporting Guideline (28).

### Outcome measures

The RSA is a self-reported scale, which consists of 33 items distributed on 6 subscales: *Perception of self* (6 items) assesses the confidence in the ability to cope with adverse events; *Planned future* (4 items) assesses the ability to set goals for the future; *Social competence* (6 items) assesses social skills and the ability to maintain relationships; *Structured style* (4 items) assesses the ability to be organized and structured; *Family cohesion* (6 items) assesses support, closeness, and security within the family; *Social resources* (7 items) assess the social network and resources outside the family (18, 23). Each item is rated on a 7-point semantic differential scale (29), e.g., “When something unfore-seen happens (I always find a solution/I often feel bewildered)”. Seventeen items are reversed to reduce acquiescence bias. A total composite score across all 33 items is computed, ranging from 33 to 231, with higher scores indicating greater levels of resilience. Each subscale score is computed as the mean score of the respective items.

The Generalized Anxiety Disorder (GAD-7) is a 7-item self-reported instrument, rated using a 4-point Likert-type scale ranging from 0 (not at all) to 3 (nearly every day) to measure anxiety-related symptoms over the past 2 weeks (30). Scores range from 0 to 21, with higher scores indicating more anxiety symptoms. An example item is “Feeling nervous, anxious or on edge”. The GAD-7 has previously showed adequate psychometric properties with a Cronbach’s alpha of 0.89 in a Norwegian population (31).

The Patient Health Questionnaire depression scale (PHQ-9) is a 9-item self-reported measure, rated using a 4-point Likert-type scale ranging from 0 (not at all) to 3 (nearly every day) to assesses symptoms of depression over the past 2 weeks (32). Scores range from 0 to 27, with higher scores indicating more depression symptoms. An example item is “Little interest or pleasure in doing things”. The PHQ-9 has previously showed adequate psychometric characteristics with a Cronbach’s alpha of 0.88 in a Norwegian population (31).

### Statistical analysis

Descriptive statistics on item and subscale scores were conducted, including means, standard deviation (SD), and range. Inter-item and item-total correlations (corrected for item overlap) were computed among all 33 RSA items and within subscales. Floor and ceiling effects were evaluated using frequencies (≥ 15%) of the lowest and highest possible scores (33). A correlation analysis was conducted among the RSA subscale scores, the RSA total score, GAD-7, and PHQ-9. All correlations were calculated using Spearman’s rank-order correlation coefficient (ρ), and interpreted as negligible (ρ < 0.10), weak (0.10 ≤ ρ < 0.30), moderate (0.30 ≤ ρ < 0.50), and strong (ρ ≥ 0.50) (34).

To evaluate unidimensionality for RSA subscales, a 1-factor measurement model was estimated for each subscale using confirmatory factor analysis (CFA). Model parameters were estimated using the weighted least-squares mean and variance adjusted (WLSMV) estimator, as the item responses on RSA are ordinal data, and compared with maximum likelihood WLSMV does not assume normality (35). Global model fit was evaluated using the adjusted χ^2^ test statistic, the root mean square error of approximation (RMSEA), the Comparative Fit Index (CFI), and the Tucker–Lewis Index (TLI). The adequacy of model fit was defined as: RMSEA ≤ 0.10, with mediocre fit in the range of 0.08–0.10, and CFI and TLI ≥ 0.95 (34). Scale reliability was evaluated using Cronbach’s alpha (α) and McDonald’s omega total (ω) for categorical variables (33, 36), with values between 0.70 and 0.95 defined as good. In the case of misfit for the measurement models on subscales, the parameter estimates, modification indices, and model residuals were examined to identify local strains of misfit and guide any model re-specifications. Adjusted models were compared with the original model using the strictly positive Satorra–Bentler scaled difference χ^2^ test statistic (37).

**Fig. 1:**
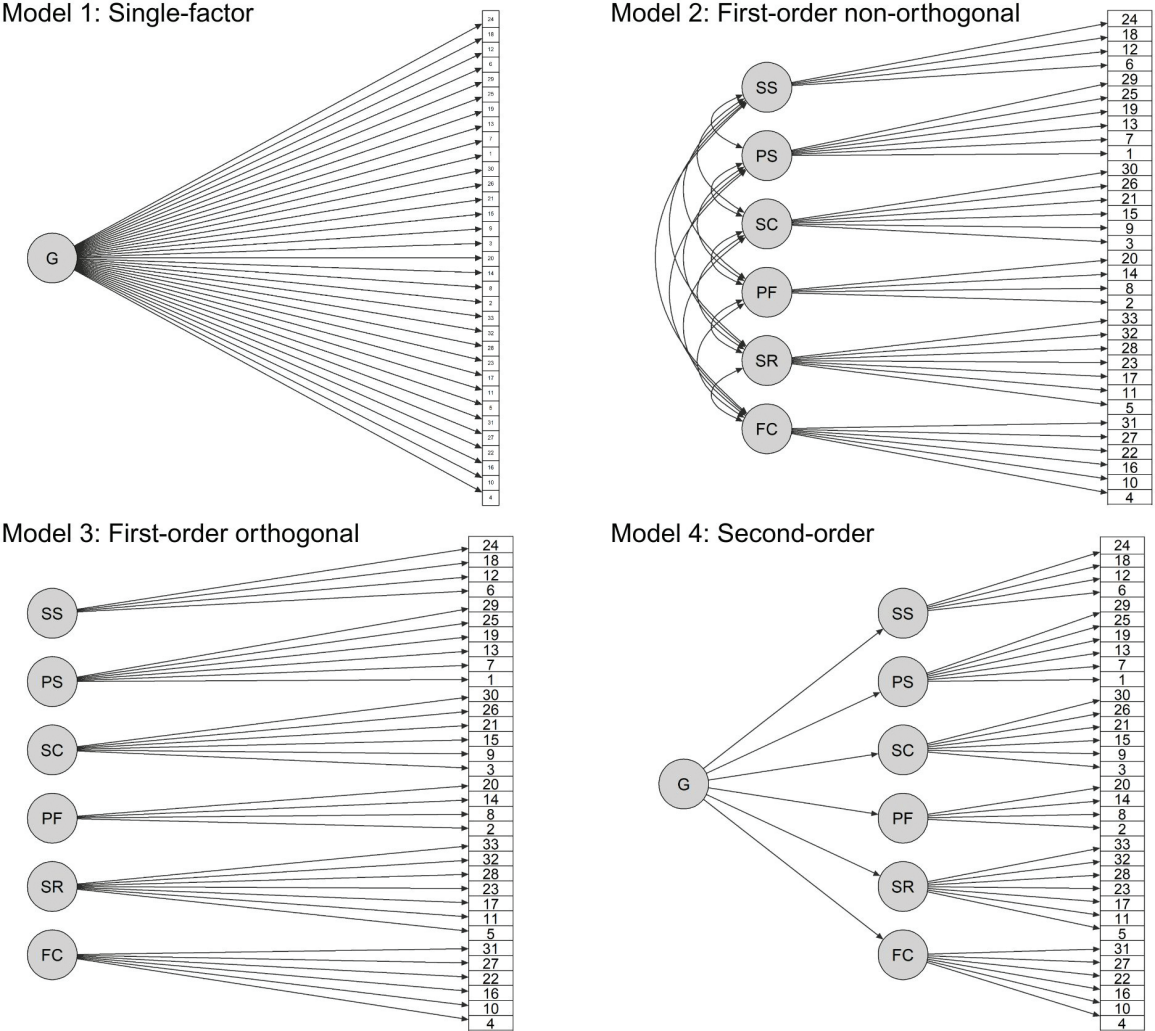
Alternative models of the factor structure of the Resilience Scale for Adults. Error terms are not shown for clarity but were included in all models.

The factor structure of the RSA was evaluated by comparing 4 different CFA models on the total scale (Fig. 1). The first model was a 1-factor model with all 33 items loading on a single factor, indicating unidimensionality (Model 1). The second model was a 6-factor model, corresponding to the 6 subscales of RSA, with correlated factors (Model 2); and the third model was an orthogonal 6-factor model with uncorrelated factors (Model 3). Finally, a second-order model was specified with all 6 factors loading on a single higher-order factor, representing a general overarching construct of resilience (Model 4). Parameters were estimated using WLSMV, and global fit statistics were compared among the models.

Analyses were conducted in R version 4.4.1 (R Foundation for Statistical Computing, Vienna, Austria) using the *psych* package for descriptive and correlation analyses, the *lavaan*, *semTools*, and *MBESS* packages for CFA. The proportion of missing data was below 1% for all measures. In accordance with recommendations for the respective outcome measures, missing data on items were handled by imputing the mean score for the GAD-7 (if 1 item is missing) and PHQ-9 (if ≤ 2 items are missing) or by the mean subscale score for the associated item in the RSA (if ≤ 3 items missing) (18, 30, 38). Due to the relatively small sample size, 7 items had response categories with no observations. Thus, a sensitivity analysis was conducted for the CFA, where response categories with 2 or less observations were collapsed. Collapsing categories with few observations did not affect interpretation of results. The data are not openly accessible due to Danish data regulations, which mandate protection of personal health information, even in anonymized form.

## RESULTS

### Psychometric properties of the RSA subscales

All subscale scores were negatively skewed and did not show any floor or ceiling effects (Table II). Three subscales (*Family cohesion*, *Planned future*, and *Perception of self*) exhibited good model fit for the 1-factor model, with significant factor loadings, and showed good reliability (Table III**,** Table SI).

**Table II:** Mean scores, range, floor and ceiling effects, and correlations of the Resilience Scale for Adults (*n* = 140)

	Variables								
	1	2	3	4	5	6	7	8	9
Correlation									
1. Family cohesion	–								
2. Social resources	0.62*	–							
3. Planned future	0.35*	0.44*	–						
4. Social competence	0.46*	0.56*	0.38*	–					
5. Perception of self	0.33*	0.39*	0.49*	0.47*	–				
6. Structured style	0.20*	0.21*	0.31*	0.14	0.25*	–			
7. RSA total score	0.71*	0.77*	0.71*	0.75*	0.73*	0.42*	–		
8. GAD-7	–0.17*	–0.17*	–0.39*	–0.30*	–0.57*	–0.15	–0.44*	–	
9. PHQ-9	–0.28*	–0.23*	–0.40*	–0.31*	–0.49*	–0.16	–0.46*	0.75*	–
Descriptive statistics									
Mean	5.51	6.03	4.61	5.02	4.93	4.75	172.41	5.26	5.51
SD	1.09	0.87	1.41	1.22	1.16	1.09	25.82	5.02	4.51
Range	1–7	1–7	1–7	1–7	1–7	1–7	93–225	0–21	0–23
Skew	–0.88	–1.55	–0.48	–0.59	–0.53	–0.22	–0.53	1.11	1.04
Floor, %	0.00	0.00	0.71	0.00	0.00	0.00	0.00	15.00	9.29
Ceiling, %	5.71	11.43	2.14	2.90	1.43	1.43	0.00	1.43	0.00

Correlation was calculated using Spearman’s rank-order correlation coefficient;

* *p* < 0.05.

**Table III:** Confirmatory factor analysis and reliability of subscales on the Resilience Scale for Adults (*n* = 140)

	Global model fit					Reliability	
	χ^2^ (df)	*p*-value	CFI	TLI	RMSEA [90% CI]	α [95% CI]	ω [95% CI]
Family cohesion	8.51 (9)	0.484	1.000	1.001	0.000 [0.000, 0.092]	0.81 [0.74, 0.85]	0.83 [0.76, 0.87]
Social resources	43.83 (14)	0.000	0.970	0.955	**0.124** [0.083, 0.166]	0.82 [0.73, 0.88]	0.83 [0.76, 0.88]
Planned future	3.68 (2)	0.159	0.997	0.992	0.078 [0.000, 0.202]	0.83 [0.76, 0.88]	0.83 [0.77, 0.88]
Social competence	30.92 (9)	0.000	0.968	**0.946**	**0.132** [0.083, 0.185]	0.79 [0.72, 0.84]	0.80 [0.70, 0.85]
Perception of self	15.15 (9)	0.087	0.986	0.977	0.070 [0.000, 0.130]	0.79 [0.71, 0.84]	0.79 [0.71, 0.84]
Structured style	7.01 (2)	0.030	**0.945**	**0.834**	**0.134** [0.036, 0.248]	**0.51** [0.35, 0.62]	**0.53** [0.36, 0.63]

Bold indicates misfit. Models were fitted to raw data using weighted least squares with mean and variance adjusted; df: degrees of freedom; CFI: comparative fit index; TLI: Tucker–Lewis index; RMSEA: root mean square error of approximation; CI: confidence interval; α: Cronbach’s coefficient alpha; ω: McDonald’s omega total.

*Social resources* did not fit the 1-factor model (see Table III). The error covariance of items 5 and 11 had the largest modification index (δ_5,11_ = 10.29) (Table SIII), indicating local dependence among these items. This was further supported by high factor loadings, λ_5_ = 0.77 and λ_11_ = 0.83, relative to the other items (λ range = 0.58 to 0.81) (see Table SI). From a conceptual perspective, the response anchors of these 2 items are very similar with regard to social relations, which is likely to be a source of local dependence: Item 5 “I can discuss personal issues with” (no one/friends and family members) and item 11, “Those who are good at encouraging me are” (some close friends and family members/no one). Freeing the constraint on δ_5,11_ improved the model fit significantly, scaled ∆χ*^2^*(1) = 19.01, *p* = < 0.001. The adjusted model exhibited good global fit, χ*^2^*(13) = 26.80, *p =* 0.013, CFI = 0.986, TFI = 0.977, RMSEA = 0.087, 90% CI (0.04,0.13), and reliability was good (ω = 0.81). The error covariance between items 5 and 11 was moderate (δ_5,11_ = 0.45, *p* < 0.001), and factor loadings for items 5 and 11, λ_5_ = 0.68 and λ_11_ = 0.77, were moderate to strong and within the overall range of factor loadings (λ = 0.59–0.83). Overall, results indicated evidence of local dependence between items 5 and 11.

*Social competence* exhibited misfit for the 1-factor model (see Table III). Factor loadings ranged from 0.51–0.89, all significant (see Table SI). The largest modification index was the error covariance between items 15 and 21 (δ_15,21_ = 12.52) (see Table SIII). The 2 items had the highest factor loadings, λ_15_ = 0.83 and λ_21_ = 0.89, compared with the other items (λ = 0.51–0.63) (Table SI). Item 15 “New friendships are something” (I make easily/I have difficulty making) and item 21 “Meeting new people is” (difficult for me/something I am good at) are conceptually related, both addressing ease or difficulty in forming new social relations, which may cause local dependency. Freeing δ_15,21_ improved model fit significantly, scaled ∆χ*^2^*(1) = 14.68, *p* = < 0.001. The adjusted model exhibited good global fit, χ*^2^*(8) = 5.69, *p =* 0.683, CFI = 1.000, TFI = 1.006, RMSEA = 0.000, 90% CI (0.00,0.08), and reliability was acceptable (ω = 0.75). The error covariance between items 15 and 21 was salient and significant (δ_15,21_ = 0.58, *p* < 0.001), and factor loadings had attenuated to λ_15_ = 0.66 and λ_21_ = 0.73. Overall, the results indicated that items 15 and 21 were locally dependent.

The 1-factor model for *Structured style* provided inadequate fit and poor reliability (see Table III). The modification indices did not indicate any single localised cause of misfit (see Table SIII). Item-total correlations within the subscale (Table SII) showed weak correlations for 2 of the 4 items, namely items 6 (ρ = 0.25) and 12 (ρ = 0.21). In addition, all average inter-item correlations were weak (ρ < 0.30) (Table SII). These findings suggest that the misfit is not likely due to any local strains, and any modifications to the model may not resolve the misfit. Hence, further analyses were terminated.

### Factor structure of the RSA

For the 4 competing CFA models on the total scale, model fit statistics are presented in Table IV. The best fitting model in the set was Model 2, i.e., the non-orthogonal model with 6 correlated factors. The superiority of Model 2 compared with the other models supports the multidimensional structure of RSA with interrelated but distinct subscales. The 6 latent factors in Model 2 were all moderately to strongly correlated with each other (range:0.39–0.79) (Table SIV). The highest correlation was between *Family cohesion* and *Social resources*, and the weakest correlation was between *Social resources* and *Structured style*. Model 3 (i.e., modelling 6 independent factors) showed the worst fit to the data, suggesting that the subscales represent related constructs. The worse fit of Model 1 and Model 4, which model a single first-order or second-order factor, respectively, provides evidence against a single overarching construct accounting for the item responses on RSA. Although Model 2 provided the best fit to data, global fit statistics indicated poor model fit with CFI and TLI values below 0.95, and none of the 4 models provided adequate fit.

**Table IV:** Confirmatory factor analysis for 1-factor, first-order, and second-order models of the Resilience Scale for Adults (*n* = 140)

	Global model fit				
	χ^2^ (df)	*p*-value	CFI	TLI	RMSEA [90% CI]
Model 1	1190.66 (495)	0.000	**0.784**	**0.770**	**0.101** [0.093, 0.108]
Model 2	768.64 (480)	0.000	**0.911**	**0.902**	0.066 [0.057, 0.074]
Model 3	2468.28 (495)	0.000	**0.388**	**0.347**	**0.169** [0.163, 0.176]
Model 4	856.80 (489)	0.000	**0.886**	**0.877**	0.074 [0.065, 0.082]

Bold indicates misfit. Models were fitted to raw data using weighted least squares with mean and variance adjusted; df: degrees of freedom; CFI: comparative fit index; TLI: Tucker–Lewis index; RMSEA: root mean square error of approximation.

As a post hoc analysis, *Structured style* was removed and correlated errors, which were identified in subscale analyses, were specified to determine the model fit of the remaining subscales for the 4 competing CFA models. This did not change the overall conclusion, as Model 2 remained the best fitting model of the set and, although improved, fit statistic remained inadequate (Table SV).

### Convergent and divergent validity

The total RSA score exhibited moderate to strong correlations with all subscales, ranging from ρ = 0.42 (*Structured style*) to ρ = 0.77 (*Social resources*). *Structured style* had weak correlations with the other subscales, except *Planned future* (ρ = 0.31). All other subscale pairs were moderately to strongly correlated, indicating that they measure related, but separate, constructs (see Table II). As expected, the RSA had weak to moderate negative correlations with GAD-7 and PHQ-9, except for *Perception of self*. This subscale was strongly associated with GAD-7 (ρ = –0.57). *Perception of self* had stronger or equally strong associations with GAD-7 and PHQ-9 than with the other subscales of RSA (ρ range from 0.25 to 0.49).

## DISCUSSION

The aim of this study was to examine the psychometric properties of the Danish version of the RSA in adults with ABI, SCI, and their family members. Three subscales, *Family cohesion*, *Planned future*, and *Perception of self*, performed well with evidence for unidimensionality. *Social resources* and *Social competence* demonstrated good reliability, but, exhibited local dependence among individual item pairs. *Structured style* did not fit the unidimensional model, and no localized model modifications were indicated to account for the misfit. The dimensional structure of the RSA was best explained by a 6-factor correlated model, indicating a multidimensional scale. No evidence was found for a single overarching factor, questioning the validity of using the total scale score.

To our knowledge, RSA has been evaluated only once in a Danish context in a sample of university students (22). In line with this study, the current study found acceptable reliability estimates (Cronbach’s alpha) for 5 subscales, and low reliability for *Structured style* in a patient and caregiver sample. Several studies have found low reliability for *Structured style*, which may indicate that the items within this subscale do not measure a single underlying construct (21, 23, 24, 39). This claim was further supported by a poor fit to the 1-factor model. Our study included a heterogenous sample, compared with previous research, which mainly included students. The comparable results beyond study samples suggest that the RSA is a robust instrument, except for *Structured style*, demonstrating consistency and enhancing generalizability.

This present study revealed misfit for *Social resources*, *Social competence*, and *Structured style*. Allowing the residual error of items to correlate improved model fit for *Social resources* and *Social competence*, but the adjusted models may not generalize beyond our study sample. In consistency with our finding, Standal et al. (21) also found misfit for *Social competence* and specified a correlation error term between items 15 and 21, suggesting local dependency between the 2 items. Hjemdal et al. (20) identified misfit for *Structured style*, with significantly different factor loadings between a Belgian and Norwegian sample, which imply variations in model fit between countries.

Results on the total RSA scale showed that Model 2, i.e., the non-orthogonal model with 6 correlated factors, demonstrated best fit compared with the alternative models. This finding supports a multidimensional scale representing separate but interrelated dimensions of resilience rather than a single uniform construct. Therefore, it might be more applicable to use the separate subscale scores rather than to summarize a total score for resilience. These findings are in alignment with results from a previous study, which found that the 6-factor structure was the best fit compared with a 1-factor model (39). However, all models on the total scale, with or without *Structured style*, showed inadequate fit in this present study.

The construct validity of the RSA was supported by the negative correlations between RSA scores and measures of anxiety and depression (GAD-7 and PHQ-9). This inverse relationship indicates that individuals with ABI, SCI, and their family members who report high resilience generally report fewer symptoms of anxiety and depression, which highlights the protective role of resilience in mental health in this mixed sample. These findings are consistent with previous studies in samples of students and individuals on long-term sick leave (21, 23). Correlations between *Perception of self* and symptoms of anxiety and depression were strong, and the overall correlations among the RSA subscales were moderate to strong, except for *Structured style*. The weak correlations for *Structured style* could be due to poor reliability and model misfit.

Overall, these findings strengthen the evidence of the validity of the RSA, also among individuals with ABI, SCI, and their family members. Implication for further research should be to identify issues regarding *Structured style*, which is necessary to improve its ability to measure the intended construct and enhance the overall validity of the RSA. Based on these results, the RSA is a multidimensional instrument, and this study provides evidence against using the total score, as the responses on RSA may not be summarized meaningfully in a single scale. Furthermore, the subscale *Structured style* should be used with caution. The remaining 5 subscales may provide a detailed account of different aspects related to resilience, which may be helpful for clinicians to identify strengths and vulnerabilities in individuals affected by ABI or SCI.

### Limitations

First, individuals with ABI or SCI and their family members were analysed collectively to obtain a sufficient sample size for CFA; however, we do not know if the scale works better or worse in one or the other of these groups. Despite the similarities they share in terms of facing life changes and disability-related challenges, ABI and SCI also differ considerably in relation to changes in cognitive, emotional, behavioural, and physical functioning, and how these life changes impact family members and their caregiver roles. These differences may affect how individuals respond to items on RSA, and any psychometric issues in one or the other group as a consequence of this may be masked by combining the samples. However, resilience is a universal concept, which is expected to be relevant and applicable across populations. The RSA was not solely intended for use in patient populations specifically, and the items are relevant no matter whether one has acquired an injury or not.

Second, the statistical measures used to estimate model fit may be influenced by the small sample size, resulting in increased uncertainty and reduced statistical power (40). For further research a larger sample size is recommended as it enables more complex models and statistical power.

Third, this study has a cross-sectional design. No information on the time perspective was available, hence test–retest reliability was not possible.

In conclusion, the present study supports the validity of the Danish version of the RSA in a heterogeneous sample of individuals with ABI, SCI, and their family members. Meanwhile, the current findings suggest that the scale score on *Structured style* should not be used for research or clinical practice. Results support the multidimensional structure of the RSA and provide evidence against using the total score across all items. Further research is recommended to replicate findings in specific subgroups of individuals with ABI or SCI or their family members or to determine measurement invariance across these populations.

## Supplementary Material


