# Supplementary material for: PSYCHOMETRIC PROPERTIES OF THE DANISH VERSION OF THE RESILIENCE SCALE FOR ADULTS IN INDIVIDUALS WITH ACQUIRED BRAIN OR SPINAL CORD INJURY, AND THEIR FAMILY MEMBERS
Source: J Rehabil Med. 2025 Oct 29;57:44078. doi: 10.2340/jrm.v57.44078 (PMC12584011; doi:10.2340/jrm.v57.44078)
Supplement: Supplementary file 1 [file JRM-57-44078-s1.pdf]

Supplementary material has been published as submitted. It has not been copyedited, or typeset by Journal of Rehabilitation Medicine

Supplementary Material

Psychometric Properties of the Danish Version of the Resilience Scale for Adults in Individuals with Acquired Brain or Spinal Cord Injury, and their Family Members

Standardized factor loadings: Table SI presents standardised factor loadings for all items in full sample (n = 140), before adjustment for model fit. Table SIV reports standardised factor loadings and factor covariance for Model 2 (non-orthogonal with six correlated factors) (n = 140).

Item descriptive statistics: Descriptive statistics in the full sample (n = 140) are provided for each item of the Resilience Scale for Adults in Table SII. Figure S1 shows the item response distribution on all items for observed cases (n = 135). Seven items had no observation for one category, one due to analysis on observed cases.

Modifications indices: Table SIII reports the modifications indices from the confirmatory factor analysis for the subscales. Indicating how to improve model fit for each subscale.

Post hoc analysis: Table SV shows the post hoc analyses with removal of *Structured style* and specifying correlated errors for the four competing CFA models.

Sensitivity analysis: 15 items included categories with two or less observations. Analyses were adjusted with collapsed categories for these items to examine effects of low frequencies in categories. The rerun of the confirmatory factor analysis for total score of the Resilience Scale for Adults with collapsed categories resulted in minor changes in fit without substantial interpretational consequences.

Table SI.

Standardized factor loadings for separate models on each subscale, N = 140

Item	Estimate	Standard Error	z	*p*
Family cohesion				
4	0.614	0.060	10.271	<0.001
10[a]	0.732	0.050	14.535	<0.001
16	0.824	0.041	19.984	<0.001
22[a]	0.630	0.047	13.338	<0.001
27	0.666	0.055	12.122	<0.001
31[a]	0.761	0.041	18.419	<0.001
Social resources				
5	0.768	0.047	16.486	<0.001
11[a]	0.834	0.043	19.184	<0.001
17	0.596	0.042	14.168	<0.001
23[a]	0.582	0.058	10.066	<0.001
28[a]	0.806	0.041	19.565	<0.001
32	0.725	0.041	17.490	<0.001
33[a]	0.643	0.055	11.732	<0.001
Planned future				
2	0.697	0.047	14.688	<0.001
8[a]	0.874	0.027	32.160	<0.001
14[a]	0.697	0.044	15.736	<0.001
20	0.794	0.040	19.980	<0.001
Social competence				
3[a]	0.506	0.069	7.348	<0.001
9	0.538	0.062	8.681	<0.001
15[a]	0.832	0.035	23.543	<0.001
21	0.890	0.032	28.155	<0.001
26[a]	0.623	0.064	9.804	<0.001
30	0.608	0.057	10.741	<0.001
Perception of self				
1	0.696	0.055	12.667	<0.001
7[a]	0.668	0.049	13.579	<0.001
13	0.696	0.054	12.988	<0.001
19[a]	0.637	0.058	11.000	<0.001
25	0.635	0.060	10.601	<0.001
29[a]	0.608	0.063	9.850	<0.001
Structured style				
6[a]	0.325	0.087	3.663	<0.001
18[a]	0.682	0.115	6.043	<0.001
12	0.261	0.087	2.774	<0.001
24	0.785	0.121	6.265	<0.001

Note. [a] = reversed items

Table SII

Item statistics on the Resilience Scale for Adults, N = 140

Item	Univariate statistics				Correlations			
					Within total scale		Within subscales	
	M	SD	Floor, %	Ceiling, %	Average inter-item	Corrected item-total	Average inter-item	Corrected item-total
Family cohesion								
4	4.89	1.68	4	18	0.18	0.46	0.38	0.51
10[a]	6.09	1.30	1	55	0.27	0.54	0.40	0.55
16	5.71	1.62	4	43	0.24	0.47	0.48	0.66
22[a]	5.66	1.32	3	31	0.25	0.50	0.40	0.54
27	5.77	1.55	3	40	0.22	0.43	0.42	0.57
31[a]	4.94	1.61	2	23	0.24	0.48	0.46	0.63
Social resources								
5	5.90	1.44	1	46	0.29	0.59	0.45	0.64
11[a]	6.31	1.16	1	61	0.27	0.54	0.46	0.65
17	5.88	1.28	1	39	0.27	0.54	0.37	0.51
23[a]	5.69	1.44	5	39	0.22	0.44	0.36	0.49
28[a]	6.28	1.20	1	60	0.27	0.54	0.44	0.63
32	6.09	1.12	1	45	0.25	0.49	0.43	0.60
33[a]	6.07	1.03	2	39	0.27	0.54	0.39	0.54
Planned future								
2	5.05	1.63	4	21	0.29	0.58	0.53	0.63
8[a]	4.46	1.84	9	16	0.25	0.50	0.59	0.73
14[a]	4.63	1.73	6	11	0.22	0.44	0.52	0.61
20	4.31	1.74	11	8	0.22	0.43	0.55	0.67
Social competence								
3[a]	4.55	1.75	4	21	0.19	0.37	0.33	0.46
9	4.90	1.71	4	22	0.25	0.50	0.35	0.48
15[a]	4.63	2.03	11	20	0.25	0.49	0.45	0.64
21	5.38	1.75	4	34	0.26	0.52	0.47	0.67
26[a]	5.62	1.60	3	39	0.21	0.41	0.38	0.53
30	5.05	1.63	3	24	0.26	0.51	0.37	0.51
Perception of self								
1	5.40	1.54	2	26	0.24	0.48	0.41	0.57
7[a]	4.87	1.45	2	11	0.28	0.56	0.41	0.58
13	5.27	1.49	3	20	0.23	0.47	0.40	0.56
19[a]	4.85	1.69	4	18	0.26	0.51	0.40	0.56
25	4.73	1.82	6	16	0.22	0.44	0.38	0.52
29[a]	4.44	1.95	9	17	0.19	0.38	0.38	0.52
Structured style								
6[a]	4.19	1.85	14	11	0.07	0.13	0.18	0.25
18[a]	4.84	1.60	2	20	0.27	0.54	0.26	0.39
12	4.74	1.83	9	19	-0.03	-0.06	0.15	0.21
24	5.21	1.57	2	24	0.16	0.32	0.27	0.41

Note. M = mean; SD = standard deviation; [a] = reversed items

Figure S1

Item response distribution on the Resilience Scale for Adults

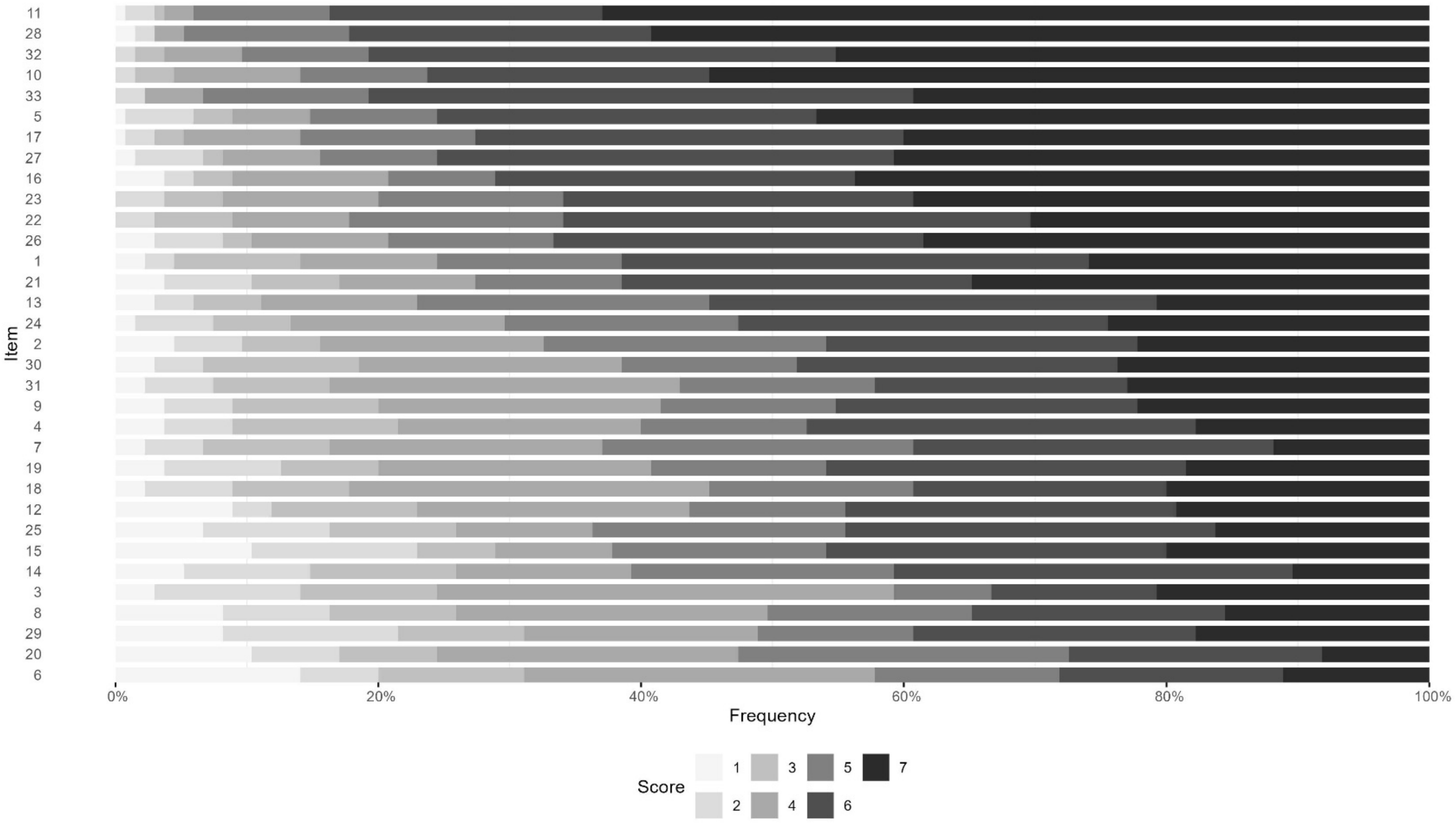

Note. N = 135, only observed cases due to imputation of mean score for five cases

Table SIII

Modification indices from confirmatory factor analyses for Social resources, Social competence, and Structured style, N = 140

Variables			Modification indices
Social resources			
item 5	~~	item 11	10.290
item 28	~~	item 32	9.522
item 5	~~	item 32	4.934
item 11	~~	item 32	4.699
item 17	~~	item 28	3.999
item 17	~~	item 23	3.208
item 11	~~	item 23	2.886
item 5	~~	item 28	1.520
item 23	~~	item 33	0.586
item 23	~~	item 32	0.398
Social competence			
item 15	~~	item 21	12.518
item 3	~~	item 9	3.090
item 3	~~	item 21	2.993
item 15	~~	item 30	2.498
item 26	~~	item 30	2.110
item 15	~~	item 26	1.803
item 9	~~	item 21	1.732
item 9	~~	item 26	1.595
item 9	~~	item 15	1.441
item 3	~~	item 30	1.404
Structured style			
item 6	~~	item 24	4.625
item 18	~~	item 12	4.625
item 6	~~	item 18	3.180
item 12	~~	item 24	3.180
item 6	~~	item 12	0.205
item 18	~~	item 24	0.205

Table SIV

Standardized factor loadings and factor covariance for Model 2 (non-orthogonal with six correlated factors), N = 140

	Family cohesion	Social resources	Planed future	Social competence	Perception of self	Structured style
Factor loadings (SE)						
4	0.651 (0.06)					
10[a]	0.794 (0.04)					
16	0.757 (0.05)					
22[a]	0.656 (0.05)					
27	0.645 (0.06)					
31[a]	0.726 (0.05)					
5		0.764 (0.05)				
11[a]		0.784 (0.04)				
17		0.694 (0.05)				
23[a]		0.613 (0.06)				
28[a]		0.749 (0.06)				
32		0.698 (0.05)				
33[a]		0.707 (0.05)				
2			0.795 (0.05)			
8[a]			0.827 (0.04)			
14[a]			0.746 (0.04)			
20			0.708 (0.05)			
3[a]				0.548 (0.07)		
9				0.696 (0.05)		
15[a]				0.768 (0.04)		
21				0.815 (0.04)		
26[a]				0.639 (0.06)		
30				0.614 (0.06)		
1					0.695 (0.05)	
7[a]					0.764 (0.05)	
13					0.673 (0.05)	
19[a]					0.636 (0.06)	
25					0.691 (0.06)	
29[a]					0.498 (0.08)	
6[a]						0.253 (0.09)*
18[a]						0.894 (0.06)
12						-0.134 (0.1)**
24						0.48 (0.072)
Factor covariance						
Family cohesion	-					
Social resources	0.786	-				
Planed future	0.414	0.534	-			
Social competence	0.553	0.665	0.514	-		

Perception of self	0.472	0.548	0.689	0.612	-	
Structured style	0.414	0.386	0.604	0.470	0.728	-

Note. All, but one estimates are statistically significant < 0.001; * p = 0.162; **p = 0.006: [a] = reversed items

Table SV

Model fit of the four competing CFA models on the RSA scale after removing Structured style and specifying correlated errors

	Global Model Fit				
	x^2 *(df)*	*p*	CFI	TLI	RMSEA [90% CI]
Model 1	910.31 (375)	0.000	**0.827**	**0.812**	**0.101**[0.093; 0.110]
Model 2	560.16 (365)	0.000	**0.937**	**0.930**	0.062 [0.052; 0.072]
Model 3	2171.45 (375)	0.000	**0.418**	**0.370**	**0.186** [0.178; 0.193]
Model 4	619.33 (370)	0.000	**0.919**	**0.911**	0.070 [0.060; 0.079]

Note. Residual errors were free to correlate among items 5 and 11 and items 15 and 21. Bold indicates misfit. Models were fitted to raw data using the weighted least squares mean and variance adjusted (WLSMV) estimator. df = degrees of Freedom; CFI = comparative fit index; TLI = Tucker-Lewis's index; RMSEA = root mean square error of approximation
